# Perceived work stress, overcommitment, balance in everyday life, individual factors, self-rated health and work ability among women and men in the public sector in Sweden – a longitudinal study

**DOI:** 10.1186/s13690-020-00512-0

**Published:** 2020-12-14

**Authors:** Carita Håkansson, Gunvor Gard, Agneta Lindegård

**Affiliations:** 1 Division of Occupational and Environmental Medicine, Lund University, Lund, Sweden; 2 Department of Health Sciences, Lund University, Lund, Sweden; 3 Institute of Stress Medicine, Region Västra Götaland, Sweden

**Keywords:** Imbalance in everyday life, Overcommitment, Work ability

## Abstract

**Background:**

The aim was to investigate whether perceived work stress, overcommiment, balance in everyday life, individual factors and self-rated health in combination predict work ability among women and men in the public sector in Sweden.

**Methods:**

A sample was randomly selected from the employee records of the participating public health care organisation in Western Sweden. In total, 2223 employees were included and answered a postal survey twice, at a 2 year interval. The survey included questions about work ability, perceived work stress, overcommitment, balance in everyday life, individual factors and self-rated health. Odds ratios with 95% confidence intervals for work ability were estimated using logistic regression.

**Results:**

Imbalance in everyday life and overcommitment predicted reduced work ability in women and imbalance in everyday life and low educational level predicted reduced work ability in men. However, when poor self-rated health was added to the models this was the strongest predictor of work ability for both genders.

**Conclusion:**

A combination of poor self-rated health, imbalance in everyday life, and overcommitment predicted reduced work ability. This multifactorial nature of work ability should be taken into account in health promotion programmes.

## Background

Stress-related or mental disorders are the most common cause of sick leave in Sweden [1]. In recent years, the sick leave rate and stress-related disorders have increased most among women working within healthcare, teaching, social work, schools, child care, and care of the elderly [2]. To promote health and prevent sick leave, it is important to explore factors predicting work ability.

Work ability is defined as the capacity to perform tasks as a function of job demands and the employee’s state of health and competence [3]. Work ability is the result of multifactorial and complex interactions, and psychosocial stressors are some of its strongest predictors [3].

One theoretical model applied to the study of psychosocial stressors is the effort-reward imbalance (ERI) model, which includes effort, reward and overcommitment [4]. This model is based on the need for reciprocity between employees’ efforts and the rewards they receive, whereby perceived stress is the outcome of an imbalance. Overcommitment is defined as “a set of attitudes, behaviors and emotions that reflect excessive striving in combination with a strong desire of being approved and esteemed” ([5], p. 55). The experience of imbalance between effort and reward will be more frequent in employees who are excessively committed to work. A longitudinal study has shown that persons with effort-reward imbalance have a higher risk of reduced work ability [6]. In concordance with this, another study has shown associations between effort-reward imbalance, overcommitment and reduced work ability [7].

A strong link was also found between self-rated health and work ability [8, 9].

The complex nature of work ability has also been highlighted in a review where associations were seen not only with classic work factors such as high physical and mental work demands, but also with more specific factors such as child care responsibilities, physical activity, educational level and age [10]. What we have been able to find, there are only two previous studies of the associations between the individual’s perception of balance in everyday life and work ability [8, 11]. One of them is a qualitative study [11] and one of them has only investigated the bivariate association between the individual’s perception of balance in everyday life and work ability [8]. Work ability is thus, as understood previously, a mixture of working life factors and life outside work [12]. However, to the best of our knowledge no study had explored whether the combination of perceived work stress, overcommitment, individual factors and balance in everyday life might predict a greater decrease in work ability compared to being exposed to just one of the above identified factors.

The aim of this study was therefore to investigate whether perceived work stress, overcommitment, balance in everyday life, individual factors and self-rated health in combination predict work ability among women and men in the public sector in Sweden.

## Method

### Study design and population

The present study is a sub-study of a longitudinal research project between 2004 and 2010 [13]. Prior to the first survey in 2004, a random sample of 5300 people was made in a region in Southern Sweden among employees in hospitals, primary care, dental care and central offices with at least 1 year of employment (at least part-time). In 2008, the study was supplemented by a new sample among employees in the same region in order to increase the proportion of men, young people and managers in the cohort. The study population in the present study included two sub-samples, one comprising persons recruited in 2004 for the original longitudinal study and who had participated in the bi-annual follow-ups until 2010, and the other recruited in 2008. Both samples were randomly selected from the employee records of the participating public organisation. In the present study, the 2008 data collection was used as a baseline and 2010 as follow-up for both samples. The total response rate was 70% (72 and 65%, respectively, in the two sub-samples). The analyses in the present study are based on a total sample of 2223, including 1773 women and 450 men.

### Measures

#### Outcome variable

*Work ability* was chosen as the outcome variable and measured with one item from the Work Ability Index (WAI) [14]. The single item is “current work ability compared with the life time best”, with 11 response alternatives from “completely unable to work” [0] to “work ability at its best” [10], i.e. the higher the score, the better the work ability. This item was dichotomised according to a median cut into low [0–7] vs. high [8–10] work ability. Using only this single item has shown a very strong relationship with the WAI (construct validity) and this item can therefore be used as a single indicator of work ability [15]. The Work Abilty Index have shown acceptable predictive validity [16], acceptable test-retest reliability [17] and good internal consistency [18].

#### Predictor variables

Predictor variables were perceived work stress, overcommiment, balance in everyday life, individual factors and self-rated health.

*Perceived work stress and overcommitment* were measured with the effort-reward imbalance questionnaire [19]. This consists of 23 items representing the three scales effort (6 items), reward (11 items) and overcommitment (6 items). The rating procedure for the items measuring effort and reward consists of two steps [19]. First, participants agree or disagree with whether or not the item content describes a typical experience of their work situation. Those who agree are asked to evaluate to what extent they usually feel distressed by this experience (from “not distressed” to “very distressed”). A sum score for these ratings is calculated for each scale. Accordingly, scores for the effort scale vary between 3 and 15 (with higher scores reflecting more stressful experiences due to high effort). Scores for the reward scale vary between 7 and 35 (with lower scores reflecting more stressful experiences due to low reward). In the present study the combined information (ratio) from the two scales (effort and reward) was used. The respondents were asked to answer the overcommitment statements (from “strongly disagree” to “strongly agree”). Overcommitment is a personal coping strategy for work stress. The sum score for this scale varies between 6 and 24, with higher scores indicating higher overcommitment [19]. Internal consistency is satisfactory and confirmatory factor analyses showed goodness of fit for the three scales [19, 20].

*Balance in everyday life* was measured with a study specific question: “How often are you pleased with your balance between the different activities in everyday life, i.e. employment, domestic work, leisure, rest/recreation and sleep?” The response alternatives were “always” [1], “often” [2] “sometimes” [3], “seldom” [4] and “never” [5]., and i.e. the higher the score, the lower the balance in everyday life.

*Individual factors* in the present study were physical activity, age, children living at home (no/yes) and educational level (university education (medium or long) vs. non university education (short education). Physical activity was measured with one question: “How physically active have you been the last three months?” [21]. Physical activity was dichotomised as mainly sedentary versus physical active (light/moderate/heavy) [21].

*Self-rated health* was measured with the question “How do you rate your health in general?” with five response alternatives from “very good” [1] to “poor” [5], i.e. the higher the score the poorer health [22]. This single item measure of self-rated health was chosen since it correlates strongly with stress and pain [23], the most common work-related disorders, indicating a high degree of construct validity [24].

### Procedure and ethics

In both the initial and the follow-up study, each presumptive participant received a covering letter, a questionnaire, a form on which to decline to participate in the study, and a stamped return envelope. It was pointed out that participation was voluntary and that confidentiality was guaranteed. Two reminders were sent out. The study was conducted according to the ethical codes and principles expressed in the Declaration of Helsinki.

### Statistical analysis

Odds ratios (OR) with 95% confidence intervals (CI) for work ability were estimated using logistic regression. We analysed the data stratified for sex due to the fact that the majority of the participants were women (80%). The great majority (87%) of the participants worked in healthcare and we therefore analysed them as one group. First bivariate analyses were done and the variables that proved to be significant were used in a multivariate analysis in two steps. In the first step all the variables, but self-rated health, were added and in step two self-rated health was added. This is because health has been shown to have a substantial effect on work ability [25]. Results were considered significant if *p* < 0.05. All the statistical analyses were performed using IBM SPSS statistics 25.

## Results

At baseline the participants were 20–66 years old with a mean age of 46 and the majority were women (80%) and employed in healthcare (87%). The majority had medium or long education (70%), had children living at home (54%), rated their health as fairly good or good (88%) and were physical active (88%). The majority did not experience effort-reward imbalance (95%), had low or medium overcommitment (78%) and about half of them rated their work ability as good (54%). More than half of them were not satisfied with their balance in everyday life (56%) (Table 1).

**Table 1:** Characteristics of the participants at baseline

Characteristic	n (%)
*Gender*	
Women	1773 (80%)
Men	450 (20%)
*Age*	
< 34 years	435 (20%)
35–44 years	491 (22%)
45–54 years	668 (30%)
55+	629 (28%)
*Educational level*	
Short education	595 (30%)
Medium or long education	1375 (70%)
*Children living at home*	
Yes	1199 (54%)
No	1020 (46%)
*Self-rated health*	
Fairly good or good	1943 (88%)
Neither good or bad, fairly bad or bad	272 (12%)
*Physical activity*	
Mostly sedentary	267 (12%)
Active	1947 (88%)
*Balance in everyday life*	
Always or often	984 (44%)
Sometimes, seldom, never	1230 (56%)
*Effort-reward imbalance*	
No	1535 (95%)
Yes	78 (5%)
*Overcommitment*	
Low	813 (38%)
Medium	874 (40%)
High	471 (22%)

### Bivariate associations between perceived work stress, overcommitment, imbalance in everyday life, individual factors, poor self-rated health and reduced work ability

In the bivariate associations between perceived work stress, overcommitment, imbalance in everyday life, individual factors, poor self-rated health and reduced work ability the associations both differ between and were the same for women and men. Associations between overcommitment, imbalance in everyday life, physical inactivity, poor self-rated health were associated with reduced work ability in women. In men an association between imbalance in everyday life, low educational level, poor self-rated health and reduced work ability were found (Table 2).

**Table 2:** Bivariate associations between perceived work stress, imbalance in everyday life, individual factors, poor self-rated health and reduced work ability among women and men

	Women			Men		
	n	B (SE)	OR (95% CI)	n	B (SE)	OR (95% CI)
High perceived work stress	1224	0.41 (0.28)	1.50 (0.87–2.60)	322	0.37 (0.48)	1.44 (0.56–3.68)
Overcommitment	1629	0.11 (0.01)***	1.12 (1.09–1.14)	422	0.03 (0.03)	1.04 (0.99–1.09)
Imbalance in everyday life	1670	0.47 (0.06)***	1.60 (1.42–1.80)	436	0.50 (0.12)***	1.65 (1.31–2.08)
Higher age	1675	−0.002 (0.01)	0.99 (0.99–1.01)	437	0.003 (0.009)	1.00 (0.99–1.02)
Low educational level	1507	0.12 (0.11)	1.13 (0.91–1.39)	362	0.64 (0.30)*	1.89 (1.06–3.39)
Physically inactive	1666	0.47 (0.15)*	1.61 (1.19–2.17)	437	0.32 (0.31)	1.38 (0.75–2.52)
Poor self-rated health	1667	0.76 (0.08)***	2.14 (1.85–2.49)	437	0.91 (0.15)***	2.48 (1.84–3.35)

*p < 0.05 **p < 0.01 ***p < 0.001

### Predictors of reduced work ability

For both women and men imbalance in everyday life was the most important predictor of reduced work ability. For women was also overcommitment a predictor of reduced work ability and for men low educational level (Table 3). When self-rated health was added to the models the odds for the other variables decreased with more than 10% and poor self-rated health became the most important predictor of reduced work ability for both women (OR 1.89, 95% CI 1.60–2.23) and men (OR 2.16, 95% CI 1.53–3.05). Furthermore, low educational level was no longer a significant predictor of reduced work ability in men.

**Table 3:** Perceived work stress, imbalance in everyday life and individual factors as predictors of reduced work ability among women (*n* = 1618) and men (*n* = 361)

	Women		Men	
	B (SE)	OR ()5% CI)	B (SE)	OR (95%CI)
Overcommitment	0.08 (0.02)***	1.08 (1.05–1.11)	–	–
Imbalance in everyday life	0.28 (0.07)***	1.33 (1.16–1.22)	0.81 (0.31)**	2.24 (1.22–4.11)
Physical inactive	0.26 (0.16)	1.29 (0.94–1.78)	–	–
Low educational level	–	–	0.49 (0.13)***	1.62 (1.27–2.08)

*p < 0.05 **p < 0.01 ***p < 0.001

## Discussion

### Principal findings

In the present study, we found that imbalance in everyday life and overcommitment predicted reduced work ability in women and imbalance in everyday life and low educational level predicted reduced work ability in men. However, when poor self-rated health was added to the models this was the strongest predictor of work ability for both genders.

### The result in relation to the results of other studies

To our knowledge this is the first study showing that imbalance in everyday life is the most important predictor of reduced work ability. However, a qualitative study (Sturesson et al., 2013) and a bivariate analysis [8] indicated that balance in everyday life is important for work ability. A possible reason why imbalance in everyday life predicted reduced work ability can be that working in the public sector in Sweden is stressful [26] and people working where have few possibilities to recovery at work [27]. Previous research has identified recovery as a mediating variable in the relationship between stressful work characteristics and poor health [28]. Imbalance in everyday life is often experienced when the possibilities for recovery both at work and outside work are reduced [29]. Furthermore, imbalance in everyday life has been shown to predict stress-related disorders both in women and men in Swedish health care [30].

The association between overcommitment and work ability suggests that women working in healthcare are so committed to their patients that their work ability is influenced. Another explanation could be the economic and organizational changes have increased in Swedish health care and have increased the external pressure to work harder, and overcommitment appears to be related to external pressures [31]. The results confirms the results of another study among food hospital service professionals [7].

Fischer’s and Martinez’s [7] results also showed associations between effort-reward imbalance and work ability [7]. However, according to Siegrist [4, 19], overcommitment is a risk factor even in the absence of effort-reward imbalance. Likewise, a review [32] showed that overcommitment without effort-reward imbalance explained the participants’ health in many studies. In the long term, overcommitment might lead to exhaustion [31]. Other studies also showed associations between overcommitment and mental health problems and leaving intentions as well as less job satisfaction [33].

Both factors at work and factors in private life, as well as the balance between them, seem to affect work ability. These results are to a certain extent in line with the work ability house model [34] but with the addition of balance in everyday life, i.e. balance between employment, domestic work, leisure, rest/recreation and sleep.

Both imbalance in everyday life and overcommitment should be regarded as warning signals and preventive measures should be taken to prevent mental or stress-related disorders, reduced work ability and sick leave.

### Strengths and limitations

One strength in the present study is the longitudinal design that makes it possible to determine the direction of causality. Another strength is that the present study was the first study to combine work factors and balance in everyday life as possible predictors of work ability.

One limitation is the fact that the selected population works in the public sector and is dominated by women. This may affect the generalisability of the results, and further studies including more men and workers from other sectors are needed.

Another limitation is that the question of balance in everyday life has not been sufficiently tested for validity and reliability. The results should therefore be interpreted with caution.

In the present study, some factors that may predict work ability were included. However, there are additional factors that predict work ability, such as body mass index, violence at work, support at work and work meaning. These factors are also important to take into consideration in future studies and when promoting work ability.

## Conclusions

A combination of poor self-rated health, imbalance in everyday life, and overcommitment predicted reduced work ability. This multifactorial nature of work ability should be taken into account in health promotion programmes.

## Data Availability

The datasets used and/or analysed during the current study are available from the corresponding author on reasonable request.

## Abbreviations

ERI: The Effort-Reward Imbalance model, which includes effort, reward and overcommitment. This model is based on the need for reciprocity between employees’ efforts and the rewards they receive, whereby perceived stress is the outcome of an imbalance. Overcommitment is defined as “a set of attitudes, behaviors and emotions that reflect excessive striving in combination with a strong desire of being approved and esteemed” ([5], p. 55)
WAI: The Work Ability Index (measures work ability)
